# Neuroinflammation in Glioblastoma: Progress and Perspectives

**DOI:** 10.3390/brainsci14070687

**Published:** 2024-07-09

**Authors:** Xin Li, Wenting Gou, Xiaoqin Zhang

**Affiliations:** Department of Pathology, School of Medicine, South China University of Technology, Guangzhou 510006, China

**Keywords:** glioblastoma, neuroinflammation, tumor microenvironment (TME), therapy

## Abstract

Glioblastoma is the most common and malignant primary brain tumor, with high morbidity and mortality. Despite an aggressive, multimodal treatment regimen, including surgical resection followed by chemotherapy and radiotherapy, the prognosis of glioblastoma patients remains poor. One formidable challenge to advancing glioblastoma therapy is the complexity of the tumor microenvironment. The tumor microenvironment of glioblastoma is a highly dynamic and heterogeneous system that consists of not only cancerous cells but also various resident or infiltrating inflammatory cells. These inflammatory cells not only provide a unique tumor environment for glioblastoma cells to develop and grow but also play important roles in regulating tumor aggressiveness and treatment resistance. Targeting the tumor microenvironment, especially neuroinflammation, has increasingly been recognized as a novel therapeutic approach in glioblastoma. In this review, we discuss the components of the tumor microenvironment in glioblastoma, focusing on neuroinflammation. We discuss the interactions between different tumor microenvironment components as well as their functions in regulating glioblastoma pathogenesis and progression. We will also discuss the anti-tumor microenvironment interventions that can be employed as potential therapeutic targets.

## 1. Introduction

Glioblastoma, also known as glioblastoma multiforme (GBM), is the most common and lethal primary brain tumor in adults, with an aggressive nature and poor treatment response [1,2]. Despite a multimodal treatment regimen including surgical resection, radiation, and chemotherapy, the prognosis of glioblastoma patients remains poor, with a median survival of only 12–15 months [3,4]. One formidable challenge in advancing glioblastoma therapy is the complexity of the tumor microenvironment (TME) [3,4,5,6]. The TME of glioblastoma is a highly dynamic and heterogeneous system that not only consists of cancerous cells but also various types of non-cancerous cells, the predominant part of which are resident or infiltrating inflammatory cells [7,8,9,10]. Over the past decades, the heterogeneous nature of glioblastoma has been extensively studied and regarded as a key factor in the poor treatment efficacy of the disease. However, most of these studies are cancer cell-centric, which may underestimate the role of the tumor microenvironment, especially neuroinflammation, in glioblastoma pathogenesis and progression.

Neuroinflammation is the inflammatory response of the brain characterized by the infiltration of various immune cells and the release of inflammation-related cytokines, chemokines, and growth factors. In glioblastoma, neuroinflammation also has the typical features of enhanced vascularization, hypoxic tumor microenvironment, and immune-suppressive milieu. All these characteristics, together with the presence of the blood–brain barrier, make the neuroinflammatory microenvironment in glioblastoma a unique pathologic process. Accumulating evidence has suggested that the neuroinflammatory microenvironment plays an important role in glioblastoma progression, invasion, and treatment response [11,12,13]. It can affect the biological behavior of tumor cells directly through inflammation–tumor cell interactions or indirectly via the release of related cytokines, chemokines, and growth factors. Neuroinflammation has increasingly been recognized as a key player and potential therapeutic target in glioblastoma [11,14,15]. A deeper understanding of the inflammatory microenvironment in glioblastoma and its interactions with the cancer cells could provide the basis for more efficient therapies.

In this review, we discuss the known and emerging concepts related to the role of the tumor microenvironment in glioblastoma carcinogenesis and progression, focusing on neuroinflammation. We discuss the components of the tumor microenvironment in glioblastoma, especially neuroinflammation-related components, and their impacts on tumor invasion and progression. We also review the anti-tumor microenvironment interventions that can potentially be employed as therapeutic targets in glioblastoma.

## 2. The Blood–Brain Barrier and Vasculature in Glioblastoma

Anatomically, the human brain is protected by a natural membrane called the blood–brain barrier (BBB), which is composed of endothelial cells connected by tight junctions and surrounded by pericytes, astrocytes, and the basement membrane [16,17]. Under normal conditions, the protective BBB has a major role in maintaining normal brain function by preventing toxins and pathogens from entering the brain through circulation [16,17]. The traditional belief even holds that the human brain is immunologically privileged since the BBB is impermeable to immune cells. This perceived dogma has recently been changed with the breakthrough findings that the human brain actually possesses a conventional and functional lymphatic system like other organs [18,19]. Moreover, it has been increasingly accepted that the integrity of BBB is actually compromised under pathological conditions, e.g., tumor-associated inflammation in glioblastoma, which leads to increased permeability of BBB and the infiltration of inflammatory cells into the brain [20,21].

These changes, together with other factors, e.g., the rapid growth of glioblastoma cells, contribute greatly to the proliferation of microvasculature, one of the most characterized pathologic features of glioblastoma [22]. A variety of angiogenic factors and chemokines have been described as being involved in the formation of vasculature in glioblastoma; the elevated level of vascular endothelial growth factor (VEGF) is regarded as the predominant one [22]. Additionally, hypoxia-inducible factor (HIF), which can actually enhance the expression of VEGF, is also reported to be a key player in vessel formation [22]. However, it is important to note that the vasculature in glioblastoma is poorly organized, hyper-dilated, and has leaky vessels. This abnormal vasculature leads to the leaking of blood components into tumor tissues and also attracts inflammatory cells, which release proangiogenic factors, thereby further enhancing the endothelial cell proliferation in glioblastoma TME.

## 3. Composition of Tumor Microenvironment in Glioblastoma

**Figure 1:**
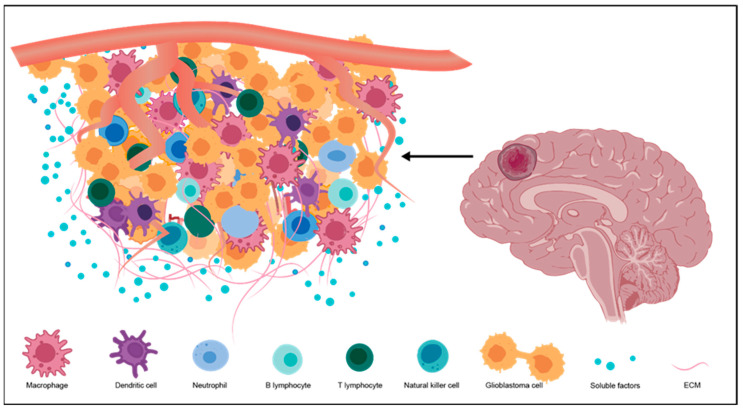
Components of the tumor microenvironment (TME) in glioblastoma.

It is now widely accepted that the tumor microenvironment (TME) of glioblastoma is a complex and dynamic system consisting of both non-cellular and cellular components [5,7,8,9,10,11,23] (Figure 1). The non-cellular components include an extracellular matrix (ECM) in which cells are embedded and various soluble factors (e.g., growth factors, cytokines, and chemokines). The cellular components include stromal cells and inflammatory cells, and inflammatory cells are predominant. It was reported that inflammatory cells in glioblastoma TME can constitute up to 30–50% of the tumor mass, which consists of resident microglia, infiltrated macrophages, and less abundant lymphoid T cells, NK cells, dendritic cells, and neutrophils. The inflammatory cells co-exist and interact with cancer cells, stromal cells, and non-cellular components, which shape the glioblastoma TME in both direct and indirect ways. The TME, especially neuroinflammation, has been regarded as the new therapeutic target for glioblastoma treatment [5,7,8,9,10,11,12,13,23,24,25].

### 3.1. Non-Cellular Components of TME in Glioblastoma

***Extracellular matrix (ECM).*** The ECM is the non-cellular component that provides an important physical scaffold for all the cellular components embedded in it. However, the ECM in the brain is unique and different from the ECM normally found in many other tissues. The ECM of the brain is largely composed of hyaluronic acid (HA), proteoglycans, and glycoproteins and demonstrates a mesh-like appearance as compared with the fibrous ECM in other tissues [26]. In the case of glioblastoma, the components of the ECM change, which can be physically reflected in the increased stiffness of TME in glioblastoma [27]. Recent studies have shown that some ECM components (e.g., HA) increase in glioblastoma tissue as compared to non-tumor tissue and contribute to the increased mobility and invasiveness of glioblastoma cells [28].

***Soluble molecular chemicals.*** For the soluble chemical components, a variety of inflammation mediators, including cytokines, chemokines, and growth factors, have been identified in the TME of glioblastoma and are involved in various signaling pathways [29]. Cytokines are signaling proteins secreted from specific immune cells, and the action modes of the cytokines include pro-inflammatory functions (e.g., IL-6, IL-8, and TNF-α) and anti-inflammatory functions (e.g., IL-4, IL-10, and TGF-β). Chemokines are small proteins that serve to mediate the migration of different cell types throughout the body. The chemokines that are highly expressed in the TME of glioblastoma have been characterized by CXCL2, IL-8, and CCL2, which can promote tumor invasion via facilitating cell proliferation, tumor growth, and angiogenesis. Additionally, tumor acidosis and low oxygen concentration are also important hallmarks of glioblastoma TME [30].

### 3.2. Cellular Components of TME in Glioblastoma

#### 3.2.1. Stromal Cells

In glioblastoma TME, the stromal cells consist of astrocytes, neurons, and vascular endothelial cells. There is growing interest in the study of glioblastomas to communicate with astrocytes and neurons [31]. Among them, astrocytes have been found to undergo reactive astrogliosis during the growth of the tumor, which could further contribute to tumor cell infiltration. These tumor-associated reactive astrocytes have also been characterized as regulating the immune environment in glioblastoma [32,33]. The neurons have also been found in the pathologic process of glioblastoma, and they may interact with glioblastoma cells via paracrine stimulation, synaptic transmission, and some other indirect means [34]. For the endothelial cells, as mentioned above, glioblastoma is one of the most vascularized malignancies with extensive endothelial cell proliferation and even the formation of glomerular structures [22,35]. Various angiogenic factors have been reported to contribute to this hallmark, including the hypoxic tumor environment, which leads to highly elevated expressions of vascular endothelial growth factor (VEGF) and, therefore, proliferation of the endothelial cells. However, these newly formed microvasculature are abnormal blood vessels and cannot provide enough blood flow and oxygen to the tumor tissue, which will accelerate the necrosis of tumor tissues, another hallmark of glioblastoma.

#### 3.2.2. Inflammatory Cells

***Microglia/Macrophages.*** Microglia and macrophages are collectively referred to as tumor-associated macrophages (TAMs), which account for 30% of the total tumor volume and are the predominant inflammatory cell populations in glioblastoma [32]. Microglial cells are the resident myeloid cells in the brain, while macrophages are infiltrating monocytes derived from the peripheral blood due to the breakdown of the BBB under pathological conditions, e.g., tumors. Microglia and macrophages have similar functions and are difficult to differentiate in most cases. However, some studies reported that they have different localization sites within the tumor tissue: resident microglia are typically found at the tumor periphery, while infiltrating macrophages tend to be more enriched in the tumor core. Recent studies reported the employment of single-cell sequencing to precisely differentiate these two cell subpopulations [36].

According to their phenotype and function, TAMs are further classified into two subtypes: the pro-inflammatory subtype (M1 macrophages) and the anti-inflammatory subtype (M2 macrophages). M1 macrophages exhibit immune-supportive and anti-tumoral functions, while M2 macrophages have immune-suppressive and pro-tumoral functions [37]. The dual function of TAMs in glioblastoma pathogenesis and progression has been a robustly debated topic in the neuroinflammation field [37,38]. The acquisition of the M1 or M2 phenotype depends on the cytokines expressed in TME. It has been found that the M1 phenotype is acquired after being stimulated with pro-inflammatory factors, such as Toll-like receptor 4 (TLR-4) ligands and interferon-gamma (IFN-γ), and then eliminates tumor cells by producing inflammatory factors (e.g., TNF-a) [39,40]. On the other hand, the M2 phenotype is triggered after receiving stimulation with anti-inflammatory factors, for example, IL-4 and IL-10. M2-phenotype TAMs show less cytotoxicity in tumor cells by producing anti-inflammatory factors (e.g., TGF-β) and are associated with promoting tumor growth. However, it should be noted that the phenotypes of TAMs are dynamic, and as the tumor progresses, the M1 and M2 phenotypes can switch to each other [30,31]. Due to their dominant number, TAMs have been regarded as promising therapeutic targets for glioblastoma treatment. The cytokines that can contribute to TAM infiltration have been characterized, including colony-stimulating factor-1 (CSF-1), CCL2, and CCL5. Moreover, multiple immunotherapy approaches that could target TAMs have been tried [37], which is detailed in the Section 4 below.

***T cells.*** T cells are also an important population of inflammatory cells in TME of glioblastoma, in spite of the fact that they constitute only a small proportion of the total cell numbers in TME (~0.25%) [41]. Of particular importance are the regulatory T cells (Tregs). Tregs are a unique population of CD4+ T cells that can regulate the overall immune homeostasis in an immunosuppressive manner [42,43]. In glioblastoma, Tregs inhibit the anti-tumor response and promote tumor-killing tolerance by secreting immunomodulatory cytokines (e.g., TGF-β and IL-10). This will, in turn, inhibit the production of anti-tumor cytokines, such as IL-2 and IFN-γ, leading to a decrease in effector cells necessary to control tumor growth. Tregs are reported to be recruited to the TME of glioblastoma by specific cytokines such as CXCR3 and CCR5, which can be secreted by glioblastoma cells and innate immune cells within the brain. In addition to Tregs, CD8+ cytotoxic T cells are another important subpopulation of T cells in glioblastoma, which can induce a tumor-killing effect and mediate tumor regression like natural killer cells. Various immunotherapy efforts have been tried to boost the cytotoxic CD8+ T cell function to treat glioblastoma, such as immune checkpoint inhibitors and CAR-T cell therapy [44,45,46]. However, these therapies are generally less efficacious in glioblastoma as compared to other malignancies due to the relatively low number of tumor-infiltrating T cells in the TME of glioblastoma.

***Natural killer cells.*** Natural killer (NK) cells have also been characterized as an important part of inflammatory cells in glioblastoma TME. Although they account for a relatively small proportion (~2% of total infiltrating inflammatory cells) like T cells, NK cells are critical for the anti-tumor immune response in glioblastoma [47]. NK cells can not only provoke tumor cell apoptosis through their direct natural cytotoxicity (e.g., granzyme B and perforin); they can also control tumor growth via secreting cytokines or regulating the activity of other inflammatory cells. For example, NK cells have been demonstrated to be able to regulate T cell-mediated immune responses by maintaining the function of dendritic cells and promoting tumor antigen presentation. On the other hand, NK cells can also be regulated by the TME. For example, glioblastoma cells express transforming growth factor (TGF-β), which can inhibit the activation of NK cell function. Glioblastoma cells can also express unique MHC-I molecules to inhibit the function of NK cells by acting as inhibitory receptor ligands [48]. Therefore, although glioblastoma is often infiltrated by NK cells, these NK cells are functionally inhibited by glioblastoma cells and TME.

***Dendritic cells.*** Dendritic cells (DCs) are a class of professional antigen-processing and presenting cells that play key roles in cancer immunity [49]. Similar to NK cells, DCs are recruited to glioblastoma via specific chemokines such as CXCL1 and CCL5. It has been shown that DCs can produce anti-tumor cytokines (e.g., IL-12), which in turn recruit more CD8+ T cells [50]. Preclinical studies have shown that the activation of DCs can improve long-term tumor survival in the mouse model of glioblastoma [50]. Clinical studies of DC vaccines in glioblastoma patients have also shown some efficacy in improving the median overall survival [50]. However, it remains to be elucidated for the standardization of DC vaccine therapy, e.g., the antigens used and the injection sites [48]. Therefore, future work on improving the efficacy of DC-based therapy in more clinical trials is needed [51].

***Neutrophil cells.*** Neutrophil cells are the most abundant population of granulocytes in the human body, which account for approximately 70% of the total number of white blood cells. In glioblastoma, neutrophils are observed to be negatively correlated with the prognosis of glioblastoma patients [52,53,54]. Neutrophils are commonly found in the center area of the glioblastoma tumor bulk and aid in tumor progression and angiogenesis. Neutrophils are attracted to the TME core by specific chemokines, e.g., CXCL8 and IL-8. They can also promote tumor proliferation and angiogenesis by secreting elastase. Recently, it was found that neutrophils are involved in the proliferation and invasion of glioblastoma cells by activating the NF-κB signaling. Additionally, there was a positive feedback loop between IL-8 expression and neutrophil infiltration into tumor sites [55]. In glioma, it was also found that the neutrophil-to-lymphocyte ratio (NLR) in the peripheral blood was positively associated with tumor grading, in which an increase in NLR may indicate a higher tumor grade and poorer patient outcomes. Moreover, compared with traditional molecular prognostic markers, e.g., IDH1 mutations, NLR can better evaluate the prognosis of glioblastoma patients and guide the treatment regimen.

## 4. Anti-TME Intervention for the Therapy of Glioblastoma

The functional role of TME, especially neuroinflammation, in the pathogenesis and tumor progression of glioblastoma makes anti-TME intervention a major novel therapy strategy for glioblastoma treatment [12,56,57,58,59]. Currently, there are two main types of TME-based therapy for glioblastoma: anti-vasculature therapy and neuroinflammation-based therapy. The latter could be further classified into four strategies: immune checkpoint inhibitors (ICIs), chimeric antigen receptor T-cell (CAR-T) therapies, vaccines, and oncolytic viruses (OVs) [57,58,59,60,61,62,63,64,65,66]. Additionally, a combined multimodal therapy of these different strategies is also extensively studied. In the following, we will provide an overview of each of these therapeutic approaches in the clinical setting for glioblastoma treatment (Table 1, Table 2, Table 3 and Table 4).

### 4.1. Anti-Vasculature Therapy

Due to the hallmark of microvascular proliferation in glioblastoma, anti-vasculature has become one of the most studied therapy approaches. A series of clinical trials have been performed to test the effectiveness of anti-vasculature therapy in glioblastoma [22,35]. The majority of these studies focus on blocking the VEGF/VEGFR signaling pathway, either through a monoclonal antibody against VEGF or with small-molecule inhibitors against VEGFR. For example, VEGF inhibition with bevacizumab, a humanized monoclonal antibody targeting VEGF, has shown effects in improving glioblastoma patients’ survival [67,68,69]. Moreover, it was found that anti-VEGF therapy can decrease vasogenic brain edema and improve blood perfusion and subsequent oxygenation, which creates conditions for better drug delivery and the efficacy of other treatments. It can also decrease the immune suppression in glioblastoma TME. Therefore, there are some strategies to combine anti-VEGF therapy with other treatment regimens, such as combining anti-vasculature therapy with immune-based approaches.

Overall, however, anti-VEGF therapy has benefitted only a subset of glioblastoma patients; the outcome in most anti-VEGF studies failed to demonstrate the benefit in patient survival [70,71,72]. There are several underlying reasons for this treatment failure. One of the major problems is the inefficient drug delivery to the tumor, which is frequent in almost all types of therapeutics for brain disease because of the BBB. Other reasons include the existence of VEGF-independent angiogenesis, such as neoangiogenesis through the CXCR4/CXCL12 axis [73]. Therefore, efforts that target angiogenesis through different action mechanisms will be helpful to increase treatment efficacy. Further analysis also revealed that the effects of anti-VEGF therapy may be dependent on the glioblastoma genetic subtypes, e.g., IDH1 mutation status, suggesting the necessity of patient subtype stratification before clinical trials [74]. Table 1 lists some of the completed phase II or III clinical trials of anti-vasculature therapy for the treatment of glioblastoma.

### 4.2. Neuroinflammation-Based Therapy

#### 4.2.1. Immune Checkpoint Inhibitors

Immune checkpoint inhibitors (ICIs) primarily refer to the monoclonal antibodies that can target the cell immune checkpoints and allow for a more robust anti-tumor effect [75]. The majority of studies on ICIs have focused on programmed death-1 (PD-1), programmed death-ligand 1 (PD-L1), and cytotoxic T-lymphocyte antigen 4 (CTLA-4), which are all important proteins of immune checkpoint pathways [76]. PD-1 is a cell surface protein of T cells and normally acts as a T cell checkpoint that keeps T cells from attacking tumor cells by binding with PD-L1. The use of PD-1 inhibitors has led to increased survival in patients with various tumor types, including glioblastoma [75,76,77]. For example, in neoadjuvant anti-PD-1 immunotherapy (pembrolizumab) in recurrent glioblastoma, satisfactory results were observed in the overall survival of patients receiving neoadjuvant pembrolizumab compared to the control group [78]. Moreover, functional activation of tumor-infiltrating T lymphocytes was observed, and interferon responses were induced in TME.

In the majority of clinical trials applying the anti-PD-1 antibody to recurrent glioblastoma patients, however, very limited efficacy in improving patients’ survival was observed. Generally, the anti-tumor effects of ICI treatment are less efficacious in glioblastoma than in other malignancies (e.g., melanoma). For example, in a phase 3 trial for a PD-1 inhibitor, although the safety of the treatment was found to be consistent with that in other tumor types, no clinical benefit was observed [79]. The scarcity of T cells in glioblastoma TME is a potential reason because the existence of infiltrating T cells in TME is fundamental to the success of ICI treatment [80]. With continuous efforts in this field, e.g., the characterization of new checkpoints and the combination of ICI treatment with other treatment regimens, the treatment efficacy of ICIs may be improved. Table 2 lists some of the completed phase II or III clinical trials of ICI therapy for the treatment of glioblastoma.

#### 4.2.2. Vaccine Therapy

Vaccine therapy utilizes one or multiple tumor-associated antigens to stimulate anti-tumor effects and has been extensively studied in multiple malignancies, including glioblastoma [81,82,83]. There are several types of tumor vaccines that are being used in cancer treatment, and the peptide-based vaccine and the dendritic cell (DC)-based vaccine are two main strategies for glioblastoma [84,85]. Peptide-based vaccines are vaccines developed based on short peptides that have epitopes with cancer cells and can act as antigenic targets to induce effective anti-tumor responses. Peptide-based vaccines have simple structures and are relatively easy to manipulate. Moreover, they have relatively lower variability as compared to other vaccines. One of the most frequently used tumor-associated antigens for peptide vaccines in glioblastoma is EGFRvIII, a deletion mutation found in approximately 20% of glioblastoma patients [84]. Dendritic cell (DC)-based vaccines employ DCs primed with whole tumor cell lysates or tumor-associated antigens to stimulate the adaptive immune system and, therefore, to control the growth of tumors. Currently, both peptide- and DC-based vaccines have been investigated in clinical trials in glioblastoma patients and represent attractive approaches for the immunotherapy of glioblastoma [82,83,84,85,86]. For example, in a phase II clinical trial of the EGFRvIII peptide vaccine study, a substantial increase in patient survival was observed [87]. For the DC-based vaccines, preclinical studies have demonstrated promising results for vaccine treatment in combination with PD-1 inhibitors. Moreover, a phase I clinical study revealed that the combination of DC-based vaccine therapy with TMZ is safe and tolerable in glioblastoma patients. Additionally, in phase III clinical trials in newly diagnosed and recurrent glioblastoma patients, it was found that autologous tumor lysate-loaded DCs could extend the patients’ survival [88].

While vaccine therapy presents an attractive method for glioblastoma therapy, disappointing results remain. In a phase III clinical trial that used the EGFRvIII peptide vaccine (rindopepimut), the results did not show a significant improvement in patient survival [89]. The potential reason is the heterogeneous and unstable expression of EGFRvIII in glioblastoma cells, which leads to the outgrowth of tumor cells that lack this antigen. Future studies are needed to reveal the effectiveness of this treatment and its impact on the overall survival of glioblastoma patients. Table 3 lists some of the completed phase II or III clinical trials of vaccine therapy for the treatment of glioblastoma.

#### 4.2.3. CAR-T Cell Therapy

CAR-T cell therapy is a novel type of immunotherapy in which T cells are modified to bind chimeric antigen receptors (CARs) to increase their ability to recognize and target tumor cells [90,91,92]. Currently, clinical trials employing CAR-T cell therapy in cancer treatment have shown safety and encouraging results, especially in hematologic malignancies. The effectiveness of CAR-T cell therapy in treating solid malignancies, e.g., glioblastoma, has also been identified [90,92,93]. There are three commonly used antigens for CAR-T cell therapy in glioblastoma: EGFRvIII, HER2, and IL-13 receptor (IL-13R) [90,91,92]. For example, a phase I clinical trial targeting EGFRvIII CAR-T cell therapy demonstrated that the intravenous administration could transfer the CAR-T cells to the brain tumor site and that the EGFRvIII level on glioblastoma tumor cells was reduced by the CAR-T cell treatment [94]. Moreover, this study also demonstrated the effects of CAR-T cell therapy on improving the immunosuppressive tumor environment, indicating the promising perspective of combinational therapy of CAR-T cell therapy with other treatment approaches. In another phase I clinical trial employing IL13R-targeted CAR-T cell therapy, it was found that the intracranial injection of CAR-T cells can achieve an anti-tumor effect in glioblastoma treatment [95]. In a phase I study employing HER2-specific CAR-T cell therapy, of the 17 recruited glioblastoma patients, only 8 demonstrated clinical benefits in overall survival [96].

As for the confounding and limited effects of CAR-T cell therapy in glioblastoma, one major problem is the heterogeneity of target-antigen expressions in tumor cells, which finally leads to heterogeneous treatment effects. Another substantial issue is how to maximize and maintain the activity of the injected CAR-T cells. It has been reported that CAR-T cell administration can induce immunosuppressive responses in the brain [94]. Therefore, successful treatment needs the development of engineered CAR-T cells that are resistant to immunosuppression. Table 4 lists some of the completed phase II or III clinical trials of CAR-T cell therapy for the treatment of glioblastoma.

#### 4.2.4. Oncolytic Virus Therapy

Oncolytic virus (OV) is able to infect cancer cells to present tumor-associated antigens and then lyse the tumor cells. Moreover, it was found that the cellular proteins released from the OV-lysed tumor cells can activate the anti-tumor immune response in multiple ways. For example, viruses can activate macrophages, and activated macrophages can enhance the infiltration of T cells into TME and, therefore, improve the immunosuppressive characteristic of glioblastoma. Therefore, OV therapy is becoming a very promising approach for the treatment of malignancies [97,98]. In glioblastoma, the effects of OV on tumor-killing have also been widely studied [98,99,100,101,102]. Multiple types of viruses are being tested for OV therapy, including retrovirus, adenovirus, herpes simplex virus, poliovirus, and measles virus [97,98]. In 2018, the recombinant oncolytic poliovirus PVSRIPO was tested in recurrent glioblastoma patients [103]. The study confirmed the potential of intratumor infusions of PVSRIPO for improving patients’ clinical outcomes. It was observed that the survival rate among patients who received PVSRIPO therapy was higher than that of historical controls.

While OV therapy has become an important focus of anti-tumor therapy, its safety and efficacy need to be tested in future research. The initial studies usually used replication-incompetent viruses to avoid complications (e.g., encephalitis), and now, an increasing number of types of viruses have been utilized as aforementioned. However, no full safety or preliminary efficacy data are currently available in the public domain. While the main goal of the current work is not to discuss each of these studies in detail, a comprehensive discussion of oncolytic virus therapy for glioblastoma can be found in several other excellent review papers [94,95,96,97]. Table 5 lists some of the completed phase II/III clinical trials of OV therapy for glioblastoma treatment.

## 5. Challenges and Perspectives

Despite encouraging results achieved so far, however, the anti-TME treatment for glioblastoma is still facing many challenges [5,58]. One of the primary impediments is the BBB. Although the BBB is compromised and more permeable during the tumor state, the anti-tumor drugs cannot cross the BBB inadequately to achieve sufficient drug accumulation in the tumor. For this reason, multiple efforts have been made to deliver pharmaceutical agents to the brain efficiently. One such important advance is focused ultrasound (FUS), which can increase the permeability of the BBB in a temporary way and enhance the delivery of drugs to the brain [104]. Currently, FUS-mediated BBB disruption has demonstrated robustness in non-invasive drug delivery to the brain and provides encouraging perspectives for the treatment of brain diseases, including glioblastoma. Another important impediment to achieving effective treatment responses, especially for immunotherapy-based approaches, is the immunosuppressive nature of glioblastoma TME. Therefore, strategies that could boost the immune response in glioblastoma TME will be helpful—for example, recruiting cytotoxic or tumor-killing inflammation cells, improving immunosuppressive properties through drugs, and transforming a ‘cold’ tumor into a ‘hot’ tumor. The third important impediment to developing effective treatment responses is the aforementioned complexity of the glioblastoma microenvironment. The enormous inter-tumor and intra-tumor heterogeneity of glioblastoma has made it one of the most difficult-to-treat malignancies in the world. Therefore, continued efforts are needed to fully understand the complex cellular and molecular components as well as their interactions involved in the TME of glioblastoma. At the same time, a synergic combination of different treatment strategies may lead to a promising curing regimen. Actually, there have been efforts to employ combinatorial therapies between immunotherapy and the current standard of care [105].

## 6. Conclusions

In summary, this review highlights the functions of neuroinflammation in glioblastoma at the cellular, molecular, and therapeutic levels. While increasing and promising results have been achieved in the anti-neuroinflammation therapy of glioblastoma, there are still many challenges. The immunosuppressive and heterogeneous characteristics of the glioblastoma microenvironment ultimately lead to resistance to anti-inflammatory therapies. Continued efforts into the tumor microenvironment will help our understanding of how these components interact with one another and contribute to the therapeutic response. This will lead to the development of more efficient and targeted therapy strategies for the treatment of glioblastoma in the future.

## Figures and Tables

**Table 1:** Clinical trials of anti-vasculature therapy for glioblastoma treatment.

	ClinicalTrials.gov ID	Target	Brief Description of the Trial	Phase	Year of Start to Completion
1	NCT01753713	VEGF	Dovitinib in treating patients with recurrent or progressive glioblastoma	2	2012–2017
2	NCT01609790	VEGF	Bevacizumab with or without trebananib in treating patients with recurrent brain tumors	2	2012–2022
3	NCT02330562	VEGF	Marizomib alone or in combination with bevacizumab in patients with recurrent glioblastoma	1 and 2	2015–2021
4	NCT02511405	VEGF	A phase 3, pivotal trial of VB-111 plus bevacizumab vs. bevacizumab in patients with recurrent glioblastoma	3	2015–2018
5	NCT02342379	VEGF	TH-302 in combination with bevacizumab for glioblastoma	2	2015–2019

Note: The data are retrieved from clinicaltrials.gov.

**Table 2:** Clinical trials of ICIs for glioblastoma treatment.

	ClinicalTrials.gov ID	Target	Brief Description of the Trial	Phase	Year of Start to Completion
1	NCT02550249	PD-1	Neoadjuvant nivolumab in glioblastoma	2	2015–2017
2	NCT02336165	PD-1	Phase 2 study of durvalumab (MEDI4736) in patients with glioblastoma	2	2015–2021
3	NCT02337491	PD-1	Pembrolizumab +/− bevacizumab for recurrent glioblastoma	2	2015–2020
4	NCT02617589	PD-1	An investigational immuno-therapy study of nivolumab compared to temozolomide, each given with radiation therapy, for newly diagnosed patients with glioblastoma	3	2016–2022
5	NCT02798406	PD-1	Combination adenovirus + pembrolizumab to trigger immune virus effects	2	2016–2021
6	NCT03018288	PD-1	Radiation therapy plus temozolomide and pembrolizumab with and without HSPPC-96 in newly diagnosed glioblastoma	2	2017–2022
7	NCT02794883	CTLA-4	Tremelimumab and durvalumab in combination or alone in treating patients with recurrent malignant glioma	2	2016–2020
8	NCT03367715	CTLA-4	Nivolumab, ipilimumab, and short-course radiotherapy in adults with newly diagnosed MGMT unmethylated glioblastoma	2	2018–2022

Note: The data are retrieved from clinicaltrials.gov.

**Table 3:** Clinical trials of vaccine therapy for glioblastoma treatment.

	ClinicalTrials.gov ID	Vaccine Type	Title	Phase	Year of Start to Completion
1	NCT00293423	Peptide vaccine	GP96 heat shock protein-peptide complex vaccine in treating patients with recurrent or progressive glioma	1 and 2	2005–2013
2	NCT00458601	Peptide vaccine	Phase II study of rindopepimut (CDX-110) in patients with glioblastoma multiforme	2	2007–2016
3	NCT00643097	Peptide vaccine	Vaccine therapy in treating patients with newly diagnosed glioblastoma	2	2007–2016
4	NCT00905060	Peptide vaccine	HSPPC-96 vaccine with temozolomide in patients with newly diagnosed glioblastoma	2	2009–2014
5	NCT01480479	Peptide vaccine	Phase III study of rindopepimut/GM-CSF in patients with newly diagnosed glioblastoma	3	2011–2016
6	NCT01920191	Peptide vaccine	Phase I/II trial of IMA950 multi-peptide vaccine plus poly-ICLC in glioblastoma	1 and 2	2013–2016
7	NCT00639639	DC vaccine	Vaccine therapy in treating patients with newly diagnosed glioblastoma	1	2006–2022
8	NCT00323115	DC vaccine	Phase II feasibility study of dendritic cell vaccination for newly diagnosed glioblastoma	2	2006–2013
9	NCT00846456	DC vaccine	Safe study of dendritic cell (DC) based therapy targeting tumor stem cells in glioblastoma	1 and 2	2009–2013
10	NCT01006044	DC vaccine	Efficacy and safety of autologous dendritic cell vaccination in glioblastoma after complete surgical resection	2	2009–2014
11	NCT01213407	DC vaccine	Dendritic cell cancer vaccine for high-grade glioma	2	2010–2015
12	NCT02465268	DC vaccine	Vaccine therapy for the treatment of newly diagnosed glioblastoma	2	2016–2023

Note: The data are retrieved from clinicaltrials.gov.

**Table 4:** Clinical trials of CAR-T cell therapy for glioblastoma treatment.

	ClinicalTrials.gov ID	Target	Brief Description of the Trial	Phase	Year of Start to Completion
1	NCT01082926	GRm13Z40-2	Phase I study of cellular immunotherapy for recurrent/refractory malignant glioma using intratumoral infusions of GRm13Z40-2, an allogeneic CD8+ Cytolitic T-Cell line genetically modified to express the IL 13-Zetakine and HyTK and to be resistant to glucocorticoids, in combination with interleukin-2	1	2010–2013
2	NCT01109095	HER2	CMV-specific cytotoxic T lymphocytes expressing CAR targeting HER2 in patients with glioblastoma	1	2010–2018
3	NCT01454596	EGFRvIII	CAR T cell receptor immunotherapy targeting EGFRvIII for patients with malignant gliomas expressing EGFRvIII	1 and 2	2012–2019
4	NCT03726515	EGFRvIII	CART-EGFRvIII + pembrolizumab in glioblastoma	1	2019–2021

Note: The data are retrieved from clinicaltrials.gov.

**Table 5:** Clinical trials of OV therapy for glioblastoma treatment.

	ClinicalTrials.gov ID	Virus Type	Brief Description of the Trial	Phase	Year of Start to Completion
1	NCT00028158	Herpes Simplex Virus	Safety and effectiveness study of G207, a tumor-killing virus, in patients with recurrent brain cancer	1 and 2	2002–2003
2	NCT00528684	Reovirus	Safety and efficacy study of REOLYSIN^®^ in the treatment of recurrent malignant gliomas	1	2006–2010
3	NCT01301430	Parvovirus	Parvovirus H-1 (ParvOryx) in patients with progressive primary or recurrent glioblastoma multiforme.	1 and 2	2011–2015
4	NCT01956734	Adenovirus	Virus DNX2401 and temozolomide in recurrent glioblastoma	1	2013–2015
5	NCT02197169	Adenovirus	DNX-2401 with interferon-gamma (IFN-γ) for recurrent glioblastoma or gliosarcoma brain tumors	1	2014–2018
6	NCT02798406	Adenovirus	Combination adenovirus + pembrolizumab to trigger immune virus effects	2	2016–2021
7	NCT03072134	Adenovirus	Neural stem cell-based virotherapy of newly diagnosed malignant glioma	1	2017–2021

Note: The data are retrieved from clinicaltrials.gov.
